# Brazilian Normative Data on Letter and Category Fluency Tasks: Effects of Gender, Age, and Geopolitical Region

**DOI:** 10.3389/fpsyg.2016.00684

**Published:** 2016-05-10

**Authors:** Izabel Hazin, Gilmara Leite, Rosinda M. Oliveira, João C. Alencar, Helenice C. Fichman, Priscila d. N. Marques, Claudia Berlim de Mello

**Affiliations:** ^1^Departmento de Psicologia, Universidade Federal do Rio Grande do NorteNatal, Brazil; ^2^Departamento de Psicologia, Universidade Federal de São PauloSão Paulo, Brazil; ^3^Instituto de Psicologia, Universidade Federal do Rio de JaneiroRio de Janeiro, Brazil; ^4^Departamento de Biociências da Universidade Federal do Rio Grande do NorteNatal, Brazil; ^5^Departamento de Psicologia, Pontifícia Universidade Católica do Rio de JaneiroRio de Janeiro, Brazil

**Keywords:** letter fluency, category fluency, dyslexia, ADH

## Abstract

Verbal fluency is a basic function of language that refers to the ability to produce fluent speech. Despite being an essentially linguistic function, its measurements are also used to evaluate executive aspects of verbal behavior. Performance in verbal fluency (VF) tasks varies according to age, education, and cognitive development. Neurodevelopmental disorders that affect the functioning of frontal areas tend to cause lower performance in VF tasks. Despite the relative consensus that has been reached in terms of the use of VF tasks for the diagnosis of dyslexia and attention-deficit/hyperactivity disorder, few studies have considered regional variations in Brazil. The present study sought to provide normative data on VF tasks in children by considering gender, age, education, and geopolitical region of origin with auxiliary purposes in neuropsychological diagnosis of disorders that occur with executive changes The study included 298 participants, 7–10 years of age of both genders, who performed three letter fluency tasks and three category fluency tasks. The data were subjected to correlational and variance analyses, with age and gender as factors. No effect of gender on the children's performance was found. However, significant differences between age groups were observed, with better performance in letter tasks in older children and better performance in letter tasks compared with category tasks. Significant regional differences in performance on the letter VF task were observed. These results reinforce the importance of regional normative data in countries with high regional cultural variations, such as Brazil.

## Introduction

Verbal fluency (VF) is a language function that is related to the ability to produce fluent speech and is typically tested in the letter and category domains (Lezak et al., 2012). VF tasks require the spontaneous generation of words according to specific criteria, under time constraints. In a letter fluency task, the subject is required to produce words that begin with specific letters (e.g., F, A, and S). In a category fluency task, the subject is required to say words that belong to specific semantic categories (e.g., animals, fruits, and clothing; Strauss et al., 2006; Moura et al., 2013).

VF tasks provide measurements of a wide range of cognitive functions, such as (1) executive function (e.g., systematic search, cognitive flexibility, and processing speed), (2) working memory and semantic memory, (3) language, and (4) verbal ability, which requires speaking and knowledge of words (Moura et al., 2015).

Performance on these tasks is particularly dependent on frontal and temporal regions of the left cerebral hemisphere, providing information about executive function and lexical skills (Perret, 1974; Miller, 1984; Ruff et al., 1997; Troyer et al., 1998; Baldo et al., 2006; Davidson et al., 2008; Birn et al., 2010). Letter fluency tasks seem to rely more on regions of the frontal cortex, whereas category fluency tasks rely more on temporal lobe functioning (Moura et al., 2015).

Studies of individuals with typical development have shown that the number of words that are produced in VF tasks is associated with changes in lexical skills and executive function that are especially related to the maturation of frontal lobe regions and may be an important marker of improvements in these functions during infancy and adolescence (Chan and Poon, 1999; Korkman et al., 2001; Riva et al., 2001; Nieto et al., 2008).

Children who are diagnosed with neurodevelopmental disorders that affect frontal areas, such as attention-deficit/hyperactivity disorder (ADHD; Barkley, 1997; Castellanos et al., 2006; Coghill et al., 2014), usually present lower performance in VF tasks compared with typically developing children (Cohen et al., 1999; Puentes-Rozo et al., 2008; Banerjee et al., 2011). Consistent with such findings, letter fluency is particularly efficient in discriminating ADHD from typical development in adolescents (Abreu et al., 2013).

In individuals with dyslexia, most studies have reported lower VF scores compared with controls (Booth et al., 2010; Lima et al., 2013; Varvara et al., 2014; Moura et al., 2015). However, although letter fluency impairment is almost always found, category fluency was shown to be preserved in some studies (e.g., Frith, 1995; Lima et al., 2013).

Such performance differences in VF are presumably attributable to impairments in executive function that are characteristic of ADHD (Brosnan et al., 2002; Lima et al., 2013; Varvara et al., 2014) and have been systematically identified in developmental dyslexia (Booth et al., 2010). Children with these clinical conditions tend to utilize different strategies when performing VF tasks. Such strategies appear to be associated with potential deficits in executive components of initiation, self-monitoring, organization, and verbal planning, as well as verbal and mnemonic skills (Silveira et al., 2009; Fernández et al., 2010; Gonçalves et al., 2013; Abreu et al., 2013; Takács et al., 2014; Gonçaves, 2015).

Despite the relative consensus that has been reached with regard to the utility of VF tasks for the diagnosis of ADHD and dyslexia, controversy has arisen in terms of the impact of sociodemographic variables on performance. Inconsistent results have been reported when the influence of gender is considered. The gender variable has been assumed by some researchers to not be influential (Riva et al., 2001; Brucki and Rocha, 2004; Hurks et al., 2010). However, other studies have suggested that gender is a significant factor, with females performing better than males (Albuquerque et al., 2007; Moura et al., 2015). Overall consensus has been reached with regard to significant improvements in performance that are observed as age and education increase (Abreu et al., 2013; Esteves et al., 2015).

Few studies have considered the impact of regional differences on performance in VF tasks. Accounting for regional differences in cognitive performance needs to go beyond the type of school or a family's socioeconomic status and should consider broad cultural population variables, such as access to information, quality of education, parents' education level, social values, and customs, among others (Ardila, 2007). In developing countries, such as Brazil, large differences are observed in children's performance on formal educational achievement tests, depending on the region of the country (Instituto Nacional de Estudos e Pesquisas Educacionais Anísio Teixeira, 2013). Such evidence suggests that normative data for neuropsychological tests in Brazil should consider cultural variables. In the clinical context, such as procedures for diagnosing neurodevelopmental disorders, such caution in sample composition may reduce the risk of false positives.

According to Mitrushina et al. (2005), normative reports should provide information about exclusion criteria with regard to sample composition and should stipulate not only conditions whose influence is well known (e.g., conditions of a psychiatric nature) but also conditions whose influence is still unclear, such as geographic region, ethnicity, and occupation. Strauss et al. (2006) stated that clinicians should be cautious when using norms that are collected from different locations because variability in demographic factors that are associated with geographic regions may interfere with performance. These authors concluded, “…local norms are therefore an asset when available” (p. 54).

The aim of the present study was to provide normative data on letter fluency (i.e., the letters F, A, and M) and category fluency (i.e., the categories animals, fruits, and clothing) in Brazilian children based on a sample of healthy children, 7–10 years of age and with different education levels and geopolitical regions of origin (southeastern, northeastern, and northern Brazil).

## Materials and methods

### Participants

The study included 298 children, 7–10 years of age who were students from private schools in the following geopolitical regions of Brazil: northeast (cities located in the states of Paraíba and Rio Grande do Norte), north (city of Belém), and southeast (city of Rio de Janeiro). All of the children belonged to socioeconomic classes C and D (based on family monthly income). Only children who were placed in regular education (elementary school) and whose first language was Portuguese were selected for the sample. Children were excluded if (1) they needed some adaptation in the curriculum to assist with their learning (e.g., due to sensory, motor, or intellectual impairments that might influence performance or due to specific learning disorders that impact reading and/or writing), (2) they presented medical conditions with neurological or psychiatric manifestations, or (3) they were using psychotropic medications. The information for establishing these criteria were collected using questionnaires that were administered to the children's parents or legal guardians.

### Materials

Three letter fluency tasks and three category fluency tasks were used. For letter fluency, the letters were “F,” “A,” and “M.” For category fluency, the categories were “animals,” “fruits,” and “clothing.” The words that were produced were recorded on protocols or tape-recorded.

### Procedures

The fluency tasks were administered in the following fixed order: letter (F, A, and M) and category (animals, fruits, and clothing). For the letter fluency tasks, the participants were instructed to say words that began with the letters F, A, and M in three separate 1-min trials. The participants were instructed not to say proper nouns or derivations of the same word. Similarly, for the category fluency tasks, the participants were instructed to generate exemplars of animals, fruits, and clothing, each within 1 min. The total number of correct words that were produced in each of the six trials was counted, according to the correction criteria that were proposed by Charchat-Fichman et al. (2011). The letter fluency score was the sum of the number of correct words in the three letter trials, and the category fluency score was the sum of the number of correct words in the three category trials. The study followed all ethical principles according to Resolution No. 466/2012 of the National Health Council that governs research with humans.

### Statistical analysis

The data were analyzed using SPSS 20 software for Windows. Homogeneity among samples regarding gender and age (categorized, 7–10 years old) was tested using the χ^2^ test of independence, and Spearman's rank correlations were used to assess associations between age and grade level. Potential main effects of the demographic variables on VF and interactions were examined using multivariate analysis of variance (MANOVA), followed by univariate analysis (ANOVA) and *post hoc* tests with Bonferroni correction. Effect sizes in the MANOVA were calculated and are presented as the partial eta-squared ($\eta_{p}^{2}$). Pearson correlation coefficients between age and VF scores and between the various VF scores were calculated. The level of statistical significance for all of the analyses was 5%.

## Results

The standardization sample in this study comprised 298 children, 7–10 years of age and residents in three Brazilian regions (southeast, northeast, and north). No difference was found between these regions in terms of the age distribution (χ^2^ = 7.24, *p* = 0.299) and gender (χ^2^ = 1.34, *p* = 0.51). Age and education level showed a strong positive correlation (ρ = 0.85, *p* < 0.001). Thus, age was elected as the variable of interest for the norms. The characterization of the participants is described in Table 1.

**Table 1:** **Demographic characterization of the normative sample**.

		**7 Years**	**8 Years**	**9 Years**	**10 Years**	**Total**
Region	Northeast	45 (50.6%)	40 (47.1%)	31 (43.7%)	15 (28.2%)	131 (44.0%)
	North	19 (21.3%)	20 (23.5%)	17 (23.9%)	16 (30.2%)	72 (24.2%)
	Southeast	25 (28.1%)	25 (29.4%)	23 (32.4%)	22 (41.5%)	95 (31.9%)
Gender	Male	35 (39.3%)	46 (54.1%)	26 (36.6%)	26 (49.1%)	133 (44.6%)
	Female	54 (60.7%)	39 (45.9%)	45 (63.4%)	27 (50.9%)	165 (55.4%)
School level	1st grade	13 (14.6%)	−	−	−	13 (100%)
	2nd grade	50 (56.2%)	15 (17.6%)	2 (2.8%)	−	67 (22.5%)
	3rd grade	23 (25.8%)	63 (74.1%)	19 (26.8%)	3 (5.7%)	108 (36.2%)
	4th grade	3 (3.4%)	7 (8.2%)	35 (49.3%)	10 (18.9%)	55 (18.5%)
	5th grade	−	−	15 (21.1%)	40 (75.5%)	55 (18.5%)
Total		89 (29.8%)	85 (28.5%)	71 (23.8%)	53 (17.7%)	298 (100%)

The MANOVA revealed effects of region (southeast, northeast, north; Pillai's trace = 0.157, *p* = 0.001, $\eta_{p}^{2}$ = 0.079) and age (7, 8, 9, and 10 years; Pillai's trace = 0.313, *p* < 0.001, $\eta_{p}^{2}$ = 0.104) on letter fluency and category fluency scores. No effect of gender was found, with no significant interactions.

Significant univariate effects of region were observed (ANOVA) for each letter: “F” [*F*_(2, 298)_ = 3.75, *p* = 0.025, $\eta_{p}^{2}$ = 0.027], “A” [*F*_(2, 298)_ = 3.89, *p* = 0.021, $\eta_{p}^{2}$ = 0.028], and “M” [*F*_(2, 298)_ = 2.99, *p* = 0.02, $\eta_{p}^{2}$ = 0.028]. An effect of region on total letter fluency was found [*F*_(2, 298)_ = 4.25, *p* < 0.015, $\eta_{p}^{2}$ = 0.03]. For the letters “F” and “M,” children from the southeastern region presented better performance, whereas children from the northeastern region presented lower performance compared with children from the other regions studied. No effect of region was found on category fluency in the three separate trials or on total category fluency (Table 2).

**Table 2:** **Comparisons of verbal fluency scores between regions**.

	**Northeast**	**North**	**Southeast**	***p* (ANOVA)**
*F*	5.4 (2.2)^a^	6.3 (2.7)^b^	6.6 (3.0)^b^	0.025
*A*	5.0 (2.2)^a^	4.9 (2.0)^a^	5.7 (2.6)^b^	0.027
*M*	5.2 (2.2)^a^	5.9 (2.3)^a, b^	6.4 (3.1)^b^	0.02
Animals	10.7 (3.2)	10.9 (2.9)	11.7 (4.2)	>0.05
Clothing	7.9 (3.1)	8.8 (2.9)	8.3 (2.6)	>0.05
Fruits	8.8 (2.6)	8.5 (2.7)	9.1 (3.7)	>0.05
Letter fluency	15.6 (5.5) ^a^	17.1 (5.2) ^a, b^	18.7 (7.5) ^b^	0.015
Category fluency	29.1 (7.9)	28.2 (6.6)	29.2 (8.6)	>0.05
Total	41.5 (11.2)^a^	45.6 (10.0)^b^	47.9 (14.2)^b^	0.002

*Different superscript letters indicate significant differences between means at the 5% level of significance (Bonferroni post hoc test)*.

**Figure 1:**
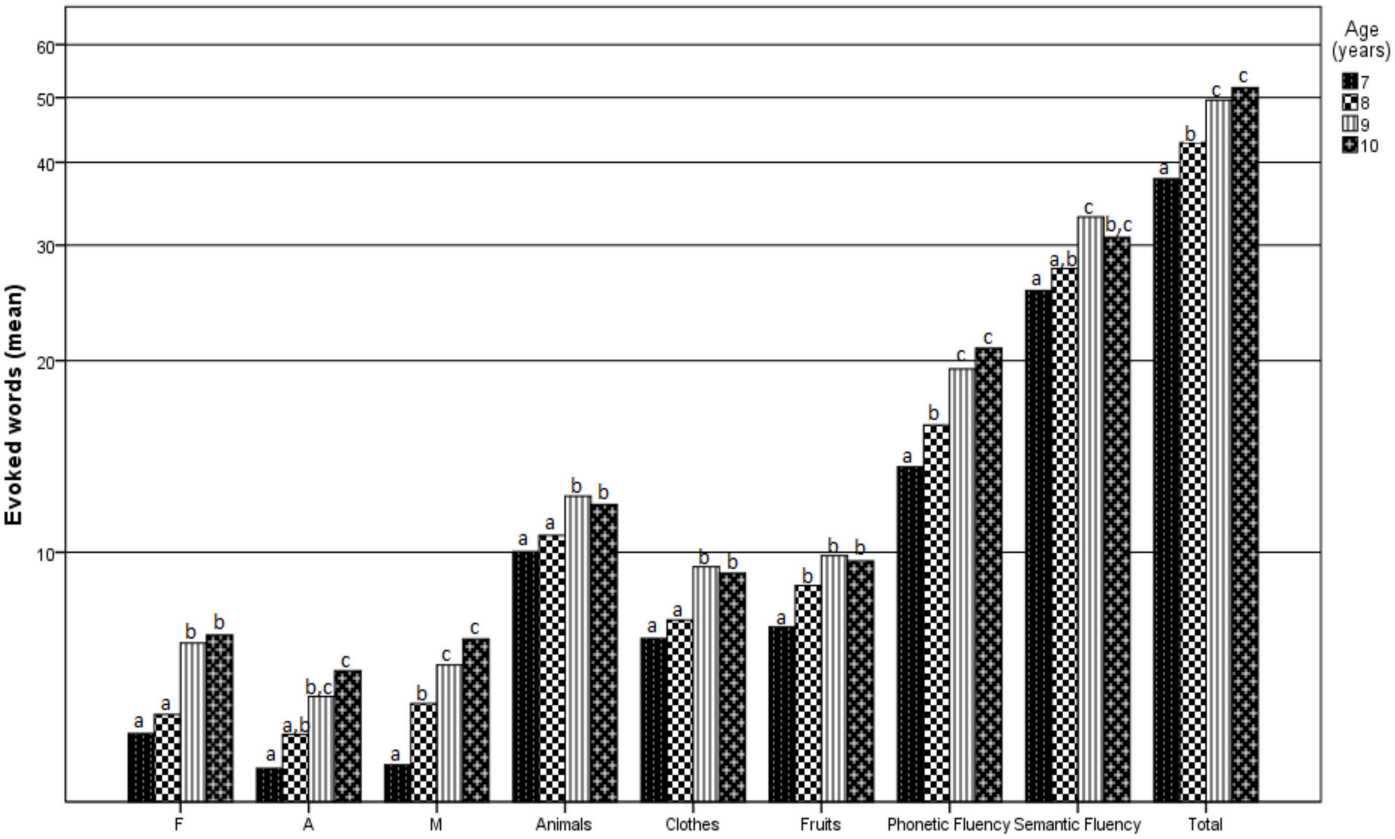
**Average number of words produced according to age and fluency task group for each letter fluency variable**. Different letters over the bars indicate significant differences between groups (Bonferroni *post hoc* test, *p* < 0.05).

The univariate ANOVA indicated that age significantly affected all letter fluency and category fluency tasks: “F” [*F*_(3, 298)_ = 17.09, *p* < 0.001, $\eta_{p}^{2}$ = 0.158], “A” [*F*_(3, 298)_ = 10.86, *p* < 0.001, $\eta_{p}^{2}$ = 0.106], “M” [*F*_(3, 298_ = 16.4, *p* < 0.001, $\eta_{p}^{2}$ = 0.152], “animals” [*F*_(3, 298)_ = 7.01, *p* < 0.001, $\eta_{p}^{2}$ = 0.071], “clothes” [*F*_(3, 298)_ = 9.71, *p* < 0.001, $\eta_{p}^{2}$ = 0.096], “fruits” [*F*_(3, 298)_ = 10.97, *p* < 0.001, $\eta_{p}^{2}$ = 0.107], letter fluency [*F*_(3, 298)_ = 23.29, *p* < 0.001, $\eta_{p}^{2}$ = 0.203], category fluency [*F*_(3, 298)_ = 14.61, *p* < 0.001, $\eta_{p}^{2}$ = 0.138], and total fluency [*F*_(3, 298)_ = 22.7, *p* < 0.001, $\eta_{p}^{2}$ = 0.199]. The Bonferroni *post hoc* test revealed significant differences between several pairs of age groups (Figure 1). Seven-year-old children underperformed 9- and 10-year-old children in the letter fluency and category fluency tasks and differed from 8-year-old children only in total letter fluency scores. Eight-year-old children underperformed 9- and 10-year-old children in total category fluency scores and total letter fluency scores. No differences were found between 9- and 10 year-old children in category fluency or letter fluency.

There were positive correlations between age and VF scores. Moderate correlations (*r* > 0.4) were found between the letter fluency variables (“F,” “A,” and “M”) and total VF scores. We also found correlations between VF variables (“F,” “A,” and “M”) and letter fluency and between semantic fluency variables (“animals,” “clothing,” and “fruits”) and category fluency, with moderate or weak correlations between letter fluency variables and semantic fluency variables (Table 3). These associations between VF variables provide evidence of the theoretical coherence of the proposed tasks.

**Table 3:** **Correlations between age and levels of letter fluency**.

	***F***	***A***	***M***	**Animals**	**Clothes**	**Fruits**	**Letter Fluency**	**Category Fluency**	**Total Fluency**
**Age**	0.360^*^	0.327^*^	0.399^*^	0.240^*^	0.300^*^	0.283^*^	0.441^*^	0.318^*^	0.438^*^
**F**		0.444^*^	0.587^*^	0.376^*^	0.285^*^	0.323^*^	0.834^*^	0.392^*^	0.682^*^
**A**			0.506^*^	0.246^*^	0.170^*^	0.289^*^	0.772^*^	0.299^*^	0.575^*^
**M**				0.356^*^	0.276^*^	0.361^*^	0.855^*^	0.390^*^	0.703^*^
**Animals**					0.386^*^	0.369^*^	0.400^*^	0.723^*^	0.679^*^
**Clothing**						0.404^*^	0.302^*^	0.668^*^	0.618^*^
**Fruits**							0.396^*^	0.693^*^	0.652^*^
**Letter Fluency**								0.442^*^	0.800^*^
**Category Fluency**									0.661^*^

*Correlations between age and levels of letter fluency*.

* *p < 0.001. The values in bold indicate moderate (0.59 > r > 0.40) and strong (r > 0.60) correlations*.

Considering that the main objective of the present study was to provide normative data, Table 4 summarizes means, standard deviations (SDs), and scores for low (–2 SD), low average (–2 to –1 SD), average (–1 to +1 SD), high average (+1 to +2 SD), and superior (> 2 SD) VF scores for each age group. Table 5 shows the means and SDs for each VF score according to age and region.

**Table 4:** **Brazilian standards for the verbal fluency test between 7 and 10 years of age**.

	***F***	***A***	***M***	**Animals**	**Clothes**	**Fruits**	**Letter Fluency**	**Category Fluency**	**Total Fluency**
**7 YEARS**									
Mean	5.0	4.3	4.4	10.0	7.2	7.6	13.7	25.6	37.8
SD	2.0	1.8	2.0	3.5	2.7	2.6	4.3	7.6	9.8
Low	0	0	0	≤ 2	≤ 1	≤ 2	≤ 5	≤ 10	≤ 18
Low Average	1–2	1–2	1–2	3–6	2–4	3–4	6–9	11–17	19–27
Average	3–7	3–6	3–6	7–13	5–9	5–10	10–18	18–33	28–47
High Average	8–9	7–8	7–8	14–17	10–12	11–12	19–22	34–40	48–57
Superior	≥10	≥9	≥9	≥18	≥13	≥13	≥23	≥41	≥58
**8 YEARS**									
Mean	7.3	6.4	7.2	11.9	9.2	9.7	20.9	30.8	51.7
SD	2.9	2.5	2.3	3.4	2.8	3.0	5.6	7.2	10.2
Low	≤ 1	≤ 1	≤ 2	≤ 5	≤ 3	≤ 3	≤ 9	≤ 16	≤ 31
Low Average	2–4	2–3	3–4	6–8	4–6	4–6	10–15	17–23	32–41
Average	5–10	4–8	5–9	9–15	7–12	7–12	16–26	24–38	42–61
High Average	11–13	9–11	10–11	16–18	13–14	13–15	27–32	39–45	62–72
Superior	≥14	≥12	≥12	≥19	≥15	≥16	≥33	≥46	≥73
**9 YEARS**									
Mean	7.1	5.8	6.5	12.3	9.5	9.9	19.4	33.1	49.6
SD	2.5	2.4	2.5	3.6	2.9	2.9	6.2	7.8	12.3
Low	≤ 2	≤ 1	≤ 1	≤ 5	≤ 3	≤ 4	≤ 6	≤ 17	≤ 24
Low Average	3–4	2–3	2–3	6–8	4–6	5–6	7–13	18–25	25–37
Average	5–9	4–8	4–9	9–15	7–12	7–12	14–25	26–40	38–61
High Average	10–12	9–10	10–11	16–19	13–15	13–15	16–31	41–48	62–74
Superior	≥13	≥11	≥12	≥20	≥16	≥16	≥32	≥49	≥75
**10 YEARS**									
Mean	7.3	6.4	7.2	11.9	9.2	9.7	20.9	30.8	51.7
SD	2.9	2.5	2.3	3.4	2.8	3	5.6	7.2	10.2
Low	≤ 1	≤ 1	≤ 2	≤ 5	≤ 3	≤ 3	≤ 9	≤ 16	≤ 31
Low Average	2–4	2–3	3–4	6–8	4–6	4–6	10–15	17–26	32–41
Average	5–10	4–8	5–9	9–15	7–12	7–12	16–26	27–38	42–61
High Average	11–13	9–11	10–11	16–18	13–14	13–15	27–32	39–45	62–72
Superior	≥14	≥12	≥12	≥19	≥15	≥16	≥33	≥46	≥73

**Table 5:** **Brazilian standards for the verbal fluency test between regions**.

		***F***	***A***	***M***	**Animals**	**Clothes**	**Fruits**	**Letter Fluency**	**Category Fluency**	**Total Fluency**
Northeast	7	4.9 ± 1.9	4.4 ± 1.8	4.2 ± 2.0	10.2 ± 3.5	6.9 ± 2.9	8.1 ± 2.4	13.5 ± 4.3	26.7 ± 8.3	37.0 ± 10.5
	8	5.3 ± 2.4	4.6 ± 1.9	5.4 ± 2.0	11.1 ± 2.5	7.8 ± 2.9	8.9 ± 2.4	15.2 ± 5.2	28.6 ± 5.0	42.0 ± 9.8
	9	5.8 ± 2.0	5.3 ± 2.3	5.6 ± 2.3	11.1 ± 2.9	9.3 ± 3.2	9.5 ± 2.8	16.7 ± 5.5	33.1 ± 8.9	43.2 ± 11.7
	10	6.7 ± 2.7	7.3 ± 2.8	6.7 ± 2.5	10.9 ± 4.3	8.7 ± 3.3	9.5 ± 3.3	20.7 ± 5.8	29.1 ± 8.2	49.9 ± 11.1
North	7	5.0 ± 2.3	4.3 ± 1.9	4.5 ± 2.0	9.7 ± 3.2	8.1 ± 2.9	6.7 ± 2.4	13.7 ± 4.1	24.5 ± 6.9	38.7 ± 8.5
	8	5.3 ± 2.5	5.3 ± 1.9	5.8 ± 2.2	10.0 ± 2.6	7.3 ± 2.2	8.0 ± 2.3	16.4 ± 4.7	25.1 ± 3.7	42.0 ± 7.7
	9	7.5 ± 2.6	5.2 ± 2.4	6.5 ± 2.2	12.4 ± 3.1	10.1 ± 2.5	9.8 ± 2.5	19.3 ± 5.8	32.4 ± 5.4	51.6 ± 9.6
	10	7.9 ± 2.5	4.9 ± 1.8	7.0 ± 2.0	11.6 ± 1.9	10.3 ± 3.0	10.0 ± 2.4	19.8 ± 4.0	31.9 ± 5.8	51.7 ± 7.5
Southeast	7	5.1 ± 1.9	4.2 ± 1.9	4.6 ± 2.2	10.0 ± 3.9	7.2 ± 2.2	7.2 ± 3.1	14.0 ± 4.6	24.4 ± 6.6	38.4 ± 9.7
	8	5.6 ± 3.3	5.2 ± 2.8	5.8 ± 3.9	10.5 ± 4.1	8.2 ± 2.9	9.4 ± 4.6	16.6 ± 8.8	28.1 ± 9.6	44.7 ± 16.5
	9	8.6 ± 2.3	6.7 ± 2.4	7.8 ± 2.6	13.9 ± 4.4	9.3 ± 2.8	10.5 ± 3.2	23.1 ± 5.7	33.6 ± 7.8	56.7 ± 10.8
	10	7.3 ± 3.3	6.8 ± 2.5	7.7 ± 2.3	12.8 ± 3.4	8.9 ± 2.2	9.5 ± 3.2	21.8 ± 6.5	31.2 ± 7.5	53.0 ± 11.5

## Discussion

The main goal of the present study was to provide normative data on performance in category and letter VF tasks in healthy Brazilian children, 7–10 years of age, adjusted for the following sociodemographic variables: gender, age, education, and region of origin. VF tasks are sensitive to different processes and cognitive abilities, particularly executive function and language, and are very relevant in both experimental and clinical contexts.

In the present study, no associations were found between performance in VF tasks (letter and category) and gender, corroborating the results of other studies (Riva et al., 2001; Brucki and Rocha, 2004; Hurks et al., 2010; Abreu et al., 2013; Moura et al., 2015). Thus, this variable was not used to stratify the normative data.

With regard to the effect of age, the findings confirmed the sensitivity of the VF tasks in identifying neurodevelopmental age markers (Matute et al., 2004; Martins et al., 2007; Moura et al., 2013). Seven-year-old children significantly underperformed 9- and 10-year-old children with regard to total letter fluency and total category fluency and differed from 8-year-old children only in the total number of words generated in the VF tasks. Eight-year-old children underperformed 9- and 10-year-old children in total category fluency scores and total letter fluency scores. No differences were found between 9- and 10-year-old children in category fluency or letter fluency, suggesting the establishment of a plateau in performance. If so, then such data would be different from those reported in some studies that considered that adequate performance in letter fluency tasks depends on frontal lobe maturation, the stabilization of which occurs around 12 years of age (Moura et al., 2013).

Based on functional imaging studies, we consider the existence of five periods of rapid growth of the frontal lobes; three of these periods can be identified in childhood. Five years of age is a milestone with regard to the first neuronal growth spurt in this area, with significant improvements in the level of attentional control processes. Additionally, the period from 7 to 9 years of age corresponds to the second growth spurt, consistent with the development of three other executive domains: information processing, cognitive flexibility, and the establishment of goals. During the third growth spurt, from 12 to 13 years of age (during the transition from childhood to adolescence), the aforementioned executive domains mature further, and executive control emerges (Diamond, 2002).

The long maturation period of the frontal cortex is one of the elements that contribute to the vulnerability to developmental disorders. The evolutionary recentness of the emergence of these abilities in humans appears to contribute equally to the susceptibility of this area to genetic and environmental variations. Consequently, a high level of comorbidity is found between alterations in executive function and the presence of invasive and global developmental disorders (e.g., autism spectrum disorder; Happe et al., 2006), maladaptive behavioral disorders (e.g., ADHD; Vital and Hazin, 2008), learning disorders (e.g., dyslexia; Gooch et al., 2011), neurological conditions (e.g., epilepsy; Luton et al., 2010), and disorders that are characterized by chromosomal alterations (e.g., Down syndrome; Lott and Dierssen, 2010).

One of the peculiarities of this study was the consideration of individual regions of origin as an independent variable. Children from northeastern Brazil presented lower performance in letter fluency compared with children from the other two regions. The participants were from three distinct regions of Brazil (northeast, north, and south), with important social and economic differences that are marked by social inequality in the country. The highest Human Development Index (HDI) are found for cities in the south and southeast of the country, whereas the northeastern cities have the worst HDI in Brazil. In the northeast region, 78% of the municipalities are characterized by low income (per capita income of R$624) and a high rate (60%) of income concentration (Instituto de Pesquisa Econômica Aplicada et al., 2013).

The impact of socioeconomic family variables and the dimensions that are considered in its rankings (e.g., parental education and family income) on the cognitive development of children, particularly regarding language and executive skills, has been reported in studies from different countries (Ardila et al., 2005; Nuru-Jeter et al., 2010; Baird et al., 2011; Sbicigo et al., 2013). Specific contexts associated with vulnerability and a lack of adequate stimulation impact the development and function of the prefrontal cortex. Although our entire sample was composed of students from private schools, these children belonged to socioeconomic classes C and D, suggesting disparities in access to different sources of information. These findings indicate that normative studies in child neuropsychology should consider the socioeconomic and cultural variability of a population when composing research samples. In the clinical context, such as procedures for diagnosing neurodevelopmental disorders, such caution in sample composition may reduce the risk of false positives.

## Author contributions

IH, GL, CB, HF, and RO make substantial contributions to conception and design, analysis and interpretation of data; GL, JA, and PM participate in acquisition of data.

## Funding

This work was funded by CNPq (Conselho Nacional de Pesquisa) and UFRN (Universidade Federal do Rio Grande do Norte).

### Conflict of interest statement

The authors declare that the research was conducted in the absence of any commercial or financial relationships that could be construed as a potential conflict of interest.
